# Success of resin infiltration treatment on interproximal tooth surfaces in young adults—A practice‐based follow‐up study

**DOI:** 10.1002/cre2.349

**Published:** 2020-11-26

**Authors:** Susanna Tiuraniemi, Jenny Yli‐Mannila, Päivi Havela, Taina Käkilehto, Hannu Vähänikkilä, Marja‐Liisa Laitala, Vuokko Anttonen

**Affiliations:** ^1^ Department of Cariology, Endodontology and Paediatric Dentistry, Research Unit of Oral Health Sciences University of Oulu Oulu Finland; ^2^ Dental Teaching Unit City of Oulu Finland; ^3^ Infrastructure for Population Studies, Faculty of Medicine University of Oulu Oulu Finland; ^4^ MRC, Oulu University Hospital and University of Oulu Oulu Finland

**Keywords:** caries prevention, interproximal initial caries lesions, resin infiltration

## Abstract

**Objectives:**

Arresting active initial caries lesions is part of the modern caries controlling system. Resin infiltration (RI) system has been found a promising method in arresting interproximal initial lesions. The aim was to investigate whether RI arrests progression of active caries lesions.

**Materials and methods:**

Participants (*n* = 20) of the retrospective study were patients in the Dental Teaching Unit, City of Oulu, Finland, mean age 26 years (SD5.2). Indication for RI was radiographically diagnosed progressing (ICDAS 1–3) interproximal lesions (*n* = 54). RI treatments were performed in 2015–2017. Controls were initial lesions in the same bitewing radiographs without RI or other treatment (*n* = 24). For analyzes teeth were categorized as lower and upper premolars and molars. The change in lesions during the follow‐up period was recorded surface wise as follows: deterioration / improvement from ICDAS score 3/improvement from ICDAS score 2/improvement per se/at follow‐up, lesion was less diffuse/no change. The changes in each tooth surface were analyzed between RI intervention and control teeth by using Chi‐square test. Proportions of successful and failed surfaces were given.

**Results:**

The mean length of follow‐up period was 17.4 m (SD7.2). Arresting of caries lesions (ICDAS scores 2 and 3) was distinctly better in RI group (63.0%) compared with the controls (29.1%). The situation progressed among 29.2% of the controls whereas the respective proportion among the intervention group was 14.8%. Progression of lesions was more distinct in all tooth groups in the control group. Outcome was successful despite the lesion depth.

**Conclusions:**

Resin infiltration seems effective in arresting progression of initial caries lesions with monitoring period of 1.5 years.

## 1 INTRODUCTION

Resin infiltration (RI) is a method where the outer surface of an initial lesion extending to dento‐enamel junction or even to outer dentin is eroded revealing its porous core. The acid found to be most efficient is 15% HCl instead of the commonly used 35% orthophosphoric acid (Neuhaus et al., 2013); HCl functions well despite the activity of the lesion. After etching the lesion is dried thoroughly for which ethanol rinsing is used. After that resin is applied on the lesion allowing the penetration of the resin by capillary phenomenon. Both interproximal and smooth surfaces can be treated.

The RI method has been used for almost two decades and there are already some systematic reviews showing arrested progression of initial caries lesions in interproximal surfaces (Doméjean et al., 2015). The method has been applied for initial caries lesions after orthodontic treatments (Gu et al., 2019). The study samples in general are small and the follow‐up periods have been maximum 3 years. There are also randomized controlled trials showing similar positive significantly better effect compared to placebo in young adults (Paris et al., 2010; Peters et al., 2018) even with 3‐year follow‐up time. The method can also be successfully used for primary teeth (Paris et al., 2010). A study on RI in showed remarkable improvement in oral health‐related quality of life among children with molar‐incisive hypomineralization (Hasmun et al., 2018).

Resin infiltration method returns the optical properties of the enamel to normal (radiolucency), which is often seen clinically (Borges et al., 2017). However strong scientific evidence is still missing (Borges et al., 2017), even if the size and colour have been found to improve compared to for example, microabrasion (Gu et al., 2019). The outcome has been found good up to 12 (Gu et al., 2019) even 24 months (Knösel et al., 2019).

The aim of the present practice‐based follow‐up study was to investigate the long‐term outcome of resin infiltration on interproximal surfaces of young adults. Another aim was to study how patients experienced the treatment.

## 2 MATERIALS AND METHODS

This is a retrospective study based on bitewing radiographs and electronic patient records as well as a survey questioning opinion of the patients on resin infiltration procedure. The study population consisted of patients who had had at least one tooth treated with resin infiltration in Dental teaching Unit, City of Oulu, Finland in 2015–2017 (*n* = 79). Treatments were performed by third to fifth year dental students of University of Oulu supervised by a clinical teacher and an author (TK).

Altogether 20 patients met the inclusion criteria: they had baseline and follow‐up X‐rays or could be reached for follow‐up X‐rays. Age and gender of the participants were recorded from the patient records. Those patients who had unclear treatment records or who did not have follow‐up radiographs taken and could not come to the appointment, were excluded. In cases, when the treatment was said to be partially failed in patient records or if the treated surface was filled or tooth was extracted, surfaces were also excluded from analyzes. Study sample comprised only interproximal lesions. For controls, lesions from the bitewing radiographs of the same individuals, that were not treated during the follow‐up time, were selected. After exclusion, the sample size was 54 surfaces (50 teeth), and the number of control surfaces 24 (23 teeth). In all cases, the follow‐up bitewing radiographs were taken at least 1 year after treatment. In some cases, there were several control radiographs (first and second year after treatment), in those cases there were two sections in the follow‐up period.

Resin infiltration was performed using a dry dam (either silicone or chemical) and if necessary, local anesthetic was applied in the region. If any suspicion of an unsuccessful RI procedure, it had been recorded in the patient records. Indication for resin infiltration had been radiographically diagnosed as active and progressing (Ekstrand et al., 2009) interproximal lesions. From baseline bitewing radiographs, control lesions without any treatment during the follow‐up period, were selected. At the time of data collection in spring 2018, some participants had already follow‐up bitewing radiographs taken. Those patients without follow‐up radiographs were invited to Dental Teaching Unit for the control bitewing radiography and to respond a questionnaire. The questionnaire consisted of questions concerning the acceptability of the duration of the RI procedure as well as absence of pain during treatment and sensitivity of the tooth afterwards. Answers to the questions were given using Visual Analogue Scale (VAS) (Luyk et al., 1988), value 1 presenting the least and value 10 the highest satisfactory option.

The baseline and the follow‐up digital bitewing radiographs were examined in two sessions by two dental students (JY and ST) and a professor of cariology (VA) in a dark room designed for observing radiographs in the teaching unit using PC monitors (Fujitsu LCD Display P24W‐7LED, 24″). The examiners were not blinded for the chronological order of the images. The monitors are evaluated frequently for maintaining their good quality.

The findings were first recorded manually and later transferred to form an electronic database for analyzes. The radiographs were approximated by Ekstrand criteria of lesion depth (1 enamel, 2 dentin‐enamel junction [DEJ], 3 dentin lesion) (Ekstrand et al., 2009) by two fourth‐year dental student. There were no deeper lesions in the study population, so class 4 was nor 5/6 were present. The classification is in line with criteria of International Caries Detection and Assessment System (ICDAS) (Ismail et al., 2007, www.iccms-web.com): 1 lesion in the enamel, 2 lesion in the DEJ = 2, 3 lesion in mid dentin = 3, class (4), 5 or 6 were not present. The baseline and follow‐up images of the lesions were compared visually, considering the size of the lesion as well how diffuse the edges were. If the treated lesions looked more diffuse (+) in the follow‐up radiograph compared to the baseline, the situation was considered progressing and respectively, if it looked less diffuse (−), the lesion was considered arrested. The three examiners investigated the radiographs together and were in unison about the results.

### 2.1 Statistics

The age of the participants was categorized (years) as follows: 15–20, 21–25, 26–30, 31–35. Distribution of lesions at baseline was given as frequencies and proportions. The change in lesions was recorded surface wise in intervention and control groups as follows: deterioration (1–2, 2–3, 3–3+, restoration), improvement from class 3 (3–2, 3–1, 3–0), improvement from class 2 or 1 (2–1, 2–0, 1–0), improvement per se, at follow‐up lesion was less diffuse, no change. In analyzes all changes in each tooth were compared between intervention and control groups by using Chi‐square test. For analyzes teeth were also categorized: lower and upper premolars, lower and upper molars. Proportions of successful and failed surfaces were given by means. The outcome by the questionnaire was presented as means (min, max). SPSS software (IBM SPSS Statistic for Windows, version 24.0. Armonk, NY: IBM Corp.) was used for the analyzes.

### 2.2 Ethics

Permission for the study was granted by the city of Oulu (OUKA/619/07.01.04.02/2017), and all participants answering the questionnaire were volunteers. No identifications were used in analyzes. No other ethical statement was needed.

## 3 RESULTS

Distribution of participants according to age and gender is presented in Table 1. The mean age was 26 years (SD 5.2). Mean length of the follow‐up period was 17.4 months (SD 7.17). The treatment was carried out on average 2.8 months (SD 2.34) after baseline x‐rays.

**TABLE 1:** Distribution of males and females in different age groups

Age (years)	Males	Females
15–20	0	4
21–25	2	0
26–30	7	3
31–35	4	0

Study sample was dominated by lower teeth (60.2%), being fairly evenly distributed among premolars and molars (Table 2). Proportion of the controls was 30.8% of the total study sample. Almost half of the samples in both groups were Ekstrand class 3 lesions; whereas proportion of class 1 was bigger in the controls than in the resin infiltration group (16.7% and 9.3%, respectively) (*p* = .649) (Table 2).

**TABLE 2:** Number of caries lesions (ICDAS scores 1–3) tooth wise before resin infiltration treatment and controls

Tooth	ICDAS 1		ICDAS 2		ICDAS 3		Total *n* (%)
	Intervention (RI)	Control	Intervention (RI)	Control	Intervention (RI)	Control	
17					1		1 (1.3)
16		1	1	1	2		5 (6.4)
15	1		2		3	1	7 (9.0)
14					4	1	5 (6.4)
24				1	2	1	4 (5.1)
25	1		3		4		8 (10.3)
26					1		1 (1.3)
27							0
37			4	2	1	1	8 (10.3)
36	1	1	1		5	3	11 (14.1)
35		1	6	1	1	2	11 (14.1)
34			1	1		1	3 (3.8)
44			1	1			2 (2.6)
45	1		3	1	1	1	7 (9.0)
46	1			1	1		3 (3.8)
47		1	1				2 (2.6)
Total n	5	4	23	9	26	11	78

**FIGURE 1:**
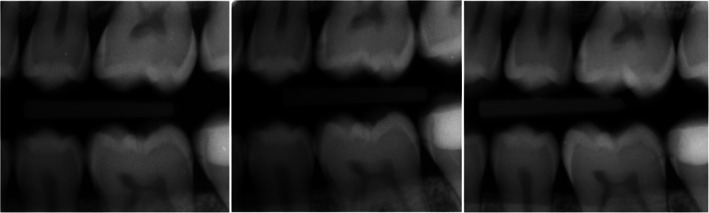
Resin infiltration treated lesions dd. 26 (mesial surface), 36 (distal surface) and 37 (mesial surface). Bite Wings taken from left to right Sep 22, 2015; Oct 3, 2016; Dec 9, 2017

Out of the 54 treated lesions only one lesion had been restored during the control time when the respective number for the controls was 2/24. Of the 32 lesions, the ICDAS class of which had remained the same, 20 were less diffuse, which can be considered arresting of the lesion. Arresting of caries lesions (Ekstrand class 1, 2 and 3) or the situation remaining unchanged was distinctly better in resin infiltration group (85.2%, 46/54) compared with the controls (70.8%, 17/24). The situation progressed among one third of the controls (29.2%) whereas the respective figure among the intervention group was 14.8% (*p* = 0.003) (Table 3). Table 4 shows how arresting of lesions occurred in different tooth types. Progression was common as for ICDAS 3, however the same was true for their arresting. Arresting of lesions occurred also among the controls especially in lower premolars. Examples of arresting in some lesions are shown in Figure 1.

**TABLE 3:** Changes in caries lesions (ICDAS scores) tooth wise in teeth treated with resin infiltration (*n* = 54 lesions) and controls (*n* = 24 lesions)

Tooth	Change in radiographic findings (ICDAS scores) during follow‐up period									
	1–2, 2–3, 3–3+, restoration		3–2, 3–1, 3–0,		2–1, 2–0, 1–0		Less diffuse, without change in category		3–3, 2–2,1–1	
	Infiltration	Control	Infiltration	Control	Infiltration	Control	Infiltration	Control	Infiltration	Control
17			1							
16	1	1	1				1			1
15	2	1	1		1				2	
14	1		1	1			1		1	
24		1	2							1
25	1		1		1		4		1	
26	1		1							
27										
37	1	1			1	1	2		1	1
36		1			1		4		1	3
35	1	1			1	1	4		1	2
34						1			1	1
44							1			1
45				1		1	2		3	
46			1			1			1	
47		1					1			
Total	8 (14.8)	7 (29.2)	9 (16.7)	2 (8.3)	5 (9.3)	5 (20.8)	20 (37.0)	0	12 (22.2)	10 (41.7)

**TABLE 4:** Distribution of arrested and progressed lesions as well as those remaining the same after resin infiltration according to depth of the lesion and tooth type compared to the controls

	Upper molars (dd. 17, 16, 26, 27)		Upper premolars (dd. 15, 14, 24, 25)		Lower molars (dd. 37, 36, 46, 47)		Lower premolars (dd. 35, 34, 44, 45)		Total	
	Intervention (RI)	Control	Intervention (RI)	Control	Intervention (RI)	Control	Intervention (RI)	Control	Intervention (RI)	Control
Progressed total	2 (33.3%)	1 (50.0%)	4 (20.0%)	2 (50.0%)	1 (7.1%)	3 (33.3%)	1 (7.1%)	1 (11.1%)	8 (14.8%)	7 (29.2%)
ICDAS 1			1			1			1	1
ICDAS 2		1	1	1		1			1	3
ICDAS 3	2		2	1	1	1	1	1	6	3
Arrested total	3 (50.0%)	0	7 (35.0%)	1 (25.0%)	3 (21.4%)	2 (22.2%)	1 (7.1%)	4 (44.4%)	14 (25.9%)	7 (29.2%)
ICDAS 1								1		1
ICDAS 2			2		2	2	1	2	5	4
ICDAS 3	3		5	1	1			1	9	2
Less diffuse total	1 (16.7%)	0	5 (25.0%)	0	7 (50.0%)	0	7 (50.0%)	0	20 (37.0%)	0
ICDAS 1			1		1				2	
ICDAS 2	1		1		3		7		12	
ICDAS 3			3		3				6	
No change total	0	1 (50.0%)	4 (20.0%)	1 (2.0%)	3 (21.4%)	4 (44.4%)	5 (35.7%)	4 (44.4%)	12 (22.2%)	10 (41.7%)
ICDAS 1		1			1	1	1		2	2
ICDAS 2			1		1		3	2	5	2
ICDAS 3			3	1	1	3	1	2	5	6
Total	6	2	20	4	14	9	14	9	54	24

Those participants who answered the questionnaire (*n* = 8) considered resin infiltration as not painful (mean VAS 8.4, min 4, max 10) as well as not causing sensitivity afterwards (mean 9.4, min 7, max 10). The duration of the procedure was well accepted (mean 8.3, min 6, max 10).

## 4 DISCUSSION

Resin infiltration showed to be effective in arresting progression of initial interproximal lesions in premolars and molars in young adults with a follow‐up period of approximately 17 months (min 7 months, max 36 months) despite the size of the lesion (Ekstrand 1–3 presenting ICDAS 1–3). The method was also quite well approved by the patients. This study gives evidence supporting use of resin infiltration technique to arrest progression of active initial caries lesions. As such it is an important tool in addition to the more traditional ones in controlling dental caries, which is beneficial both for the patient and for the society.

Overall proportion of the arrested resin infiltrated lesions was distinctly higher than that in the control group. Arresting took place in all ICDAS categories. The results are in accord with previous findings showing arresting capability of the method. In the 3‐year follow‐up study by Meyer‐Lueckel et al. (2012) on 19 young adults, 3.8% of the treated lesions had progressed, when the respective figure for the controls was 34.6% (Meyer‐Lueckel et al., 2012). Similar results were reported by Martignon et al. (2012) in their split‐mouth placebo‐controlled randomized clinical trial, with the so far biggest study‐population and longest follow‐up time. Their outcome was that in 3 years 32% of the lesions treated with infiltration, 41% of the sealed and 70% of the placebo surfaces had progressed. There was a tendency that lesions extending to dentin progressed the most (Martignon et al., 2012).

It is common to give individual instructions for home care (Paris et al., 2010), as was the case here as well. Patients with initial lesions have instructions for flossing in Dental Teaching Unit. This might explain why arresting of lesions was commonly discovered also among the controls. That or any other behavioral factors were not included in analyzes. This is most important to control the situation and prevent development of future lesions. In other studies fluoride varnish (Ekstrand et al., 2010) and sealing (Martignon et al., 2012) have been used for controls, yet, the outcome has favored resin infiltration. Here arresting of lesions was common in lower teeth.

The environment of the lesion may affect the outcome by resin infiltration. Ferreira et al. (2018) have suggested that amount of organic material in the environment of the developing lesion deteriorates its arresting capability by resin infiltration. The lesion may progress, even if the common problem with conventional restorations, leakage is not seen. Careful cleaning using pumice is necessary before resin infiltration. By treating early caries lesions with resin infiltration in caries active individuals we can prevent the circle of repetitive restorative treatments (Forss & Widström, 2003, Martignon et al., 2012). Caries activity was not assessed in the present study.

The sample size in this study was similar with other respective studies. If they were invited for the follow‐up X‐rays, patients got a concise examination of their dentition for free, which was aimed to increase the number of participants. However, all of them could not be reached, in most cases they had moved out of Oulu or it was not possible to contact them for any reason. Cariological status of the individuals would be an interesting background information in future studies, but unfortunately is not available. However, resin infiltration is indicated in arresting progressive initial lesions, which again indicate caries activity.

The number of control lesions was limited because lesions seen in the radiographs were often either treated with resin infiltration or filled. Some treatment records stated that the procedure had partially failed most often due to failed moisture control. Those lesions were not included in the study sample. Some lesions had to be excluded because they were technically near the edge of the bitewing images (especially concerning second molars and first premolars). In some cases, the quality of baseline images was too poor for evaluations or there were too big differences (for instance in projection or quality) between baseline and control images which made lesion comparison difficult. Not blinding the examiners of the images as for chronological order of the images or whether they represented treated or control sites, can also be considered as a weakness.

The mean age of the participants was approximately the same as in other studies. The method seems appropriate for the young, indeed there are no studies for example, considering root caries of the elderly. Data on treated white spot lesions were not available here. In addition to lecturers, dental students performed the resin infiltration treatments. This proves that the method can be carried out successfully by junior dentists and most likely also by oral hygienists.

The factors making the procedure challenging are separation of teeth, control of moisture, location of the tooth, gagging and drooling. Sometimes local anesthesia is needed. Separation bands could be helpful. These preparations can make RI time consuming. The time needed for one infiltration is approximately the same as for a small filling. The method can be considered costly, however in estimating the costs, the longevity of the infiltrated surface should be kept in mind. If it is successful and needs not to be redone.

Only those participants who came for the follow‐up visit due to the radiographs answered the questionnaire, therefore, the sample was small (*n* = 8). The ones who responded considered the method pleasant (no pain, not too time‐consuming, no sensitivity afterwards). The only reported side‐effect was bad tastes during the procedure. The feed‐back on the method should be asked a few days after the treatment.

## 5 CONCLUSION

Resin infiltration seems to be a clinically effective method in arresting early carious lesions, especially for lesions in ICDAS classes 1 to 2 but even for 3. In estimating changes in caries lesions over time their size remaining the same or them becoming less diffuse, are signs of arresting. Out of the infiltrated lesions, a noticeable proportion, here, seemed to be less diffuse after the follow‐up period. In controlling caries mini‐invasive procedures are more acceptable than invasive, which favors the use of RI over restorative treatment.

## CONFLICT OF INTEREST

The authors declare that they have no conflict of interests.
